# Exploring the cellular basis of human disease through a large-scale mapping of deleterious genes to cell types

**DOI:** 10.1186/s13073-015-0212-9

**Published:** 2015-09-01

**Authors:** Alex J. Cornish, Ioannis Filippis, Alessia David, Michael J.E. Sternberg

**Affiliations:** Department of Life Sciences, Imperial College London, Exhibition Road, London, SW7 2AZ UK

## Abstract

**Background:**

Each cell type found within the human body performs a diverse and unique set of functions, the disruption of which can lead to disease. However, there currently exists no systematic mapping between cell types and the diseases they can cause.

**Methods:**

In this study, we integrate protein–protein interaction data with high-quality cell-type-specific gene expression data from the FANTOM5 project to build the largest collection of cell-type-specific interactomes created to date. We develop a novel method, called gene set compactness (GSC), that contrasts the relative positions of disease-associated genes across 73 cell-type-specific interactomes to map genes associated with 196 diseases to the cell types they affect. We conduct text-mining of the PubMed database to produce an independent resource of disease-associated cell types, which we use to validate our method.

**Results:**

The GSC method successfully identifies known disease–cell-type associations, as well as highlighting associations that warrant further study. This includes mast cells and multiple sclerosis, a cell population currently being targeted in a multiple sclerosis phase 2 clinical trial. Furthermore, we build a cell-type-based diseasome using the cell types identified as manifesting each disease, offering insight into diseases linked through etiology.

**Conclusions:**

The data set produced in this study represents the first large-scale mapping of diseases to the cell types in which they are manifested and will therefore be useful in the study of disease systems. Overall, we demonstrate that our approach links disease-associated genes to the phenotypes they produce, a key goal within systems medicine.

**Electronic supplementary material:**

The online version of this article (doi:10.1186/s13073-015-0212-9) contains supplementary material, which is available to authorized users.

## Background

Identifying the cell types that contribute to the development of a disease is key in understanding its etiology. It is estimated that there are at least 400 different cell types present within the human body [1], each performing a unique repertoire of functions, the disruption of which may lead to the development of a disease [2]. Thousands of genes that influence human disease have been identified through linkage analysis, genome-wide association studies and genome sequencing [3]. In many cases, the cell types that these genes directly affect and through which promote disease development have yet to be characterized or are still being debated. Identification of these cell types will further our understanding of the genetic basis of these diseases and the underpinning molecular pathways and processes. In this study, we refer to the cell types directly affected by the disease-associated genes as the disease-manifesting cell types.

Large-scale mappings have previously identified associations between diseases [4], genes [5] and tissues [6]. However, there currently exists no large-scale mapping of diseases to the cell types in which they are manifested. Developments in gene expression profiling technology have led to the availability of tissue- and cell-type-specific gene expression data [7–9], which have been integrated with known disease-associated genes to identify systematically associations between diseases, tissues [10] and a limited number of cell types [11]. However, a lack of high-quality cell-type-specific gene expression data has previously limited the large-scale mapping of diseases to cell types.

The molecular basis of diseases can also be explored using the interactome, a network created by integrating all interactions known to occur between proteins. Tens of thousands of protein–protein interactions (PPIs) have been identified [12] and used in tasks such as the prioritization of disease-associated genes [13, 14] and the prediction of the phenotypic impact of single amino acid variants [15]. However, the majority of methods that detect PPIs operate in vitro, meaning that unlike gene expression, we have little understanding of the contexts in which PPIs take place. This lack of context-specific PPI data means that the majority of methods that use the interactome to explore the molecular basis of a disease use a generic PPI network [13, 14], rather than a PPI network specific to the context of the disease being studied. This has been seen to limit the success of these methods [16]. Computational approaches have been developed to create context-specific biological networks [16–21]. These approaches often use gene expression data to modify generic PPI networks, either through the removal of proteins not expressed in a given context [16–18, 20] or through the re-weighting of interactions deemed more likely to occur in a given context [16]. Whilst these methods have been used to create tissue-specific interactomes, few cell-type-specific interactomes have been created.

In this study, we integrate high-quality cell-type-specific gene expression data and PPI data to build a collection of 73 cell-type-specific interactomes and use these interactomes to create the first large-scale mapping of diseases to cell types. We use gene expression data from the FANTOM5 project [8], which represents the largest atlas of cell-type-specific gene expression produced to date. These data were created using primary cell samples rather than immortalized cell lines, resulting in higher-quality gene expression profiles [8]. By comparing the clustering of sets of disease-associated genes across these cell-type-specific interactomes, we demonstrate that it is possible to use cell-type-specific interactomes to identify the cell types in which a disease is most likely to be manifested. This approach is validated using text-mined disease–cell-type associations from the PubMed database. An implementation of the method described in this study and the 73 cell-type-specific interactomes are available to download [22, 23]. These resources will be useful in the identification of additional disease-associated cell types as more gene expression data become available, as well as in the development of tools better able to explore the etiology of a disease given its cellular context. Using this method, we identify known disease–cell-type associations and associations that warrant further study.

## Methods

### Method implementation and data availability

We have developed an R package called DiseaseCellTypes that contains implementations of the gene set compactness (GSC) and gene set overexpression (GSO) methods and the method used to create the cell-type-specific interactomes. DiseaseCellTypes is available to download from [22]. This package also includes the data and a vignette containing the code required to reproduce the results detailed in this study. The 73 cell-type-specific interactomes are available to download from [23].

### The DisGeNET database

Sets of disease-associated genes were obtained from the DisGeNET database (v2.1) [3], which integrates expertly curated associations, associations predicted using animal models and associations identified using automated text-mining (Additional file 1: Table S1). In total, DisGeNET contains 13,185 diseases associated with one or more genes. We completed a number of filtering steps to extract the highest-quality associations between human diseases and genes, reducing the number of diseases associated with one or more genes to 1544.

We first removed associations predicted using animal models, associations identified using automated text-mining and associations related to the genetic response to environmental chemicals, as we deemed these associations less likely to be of high quality and less relevant to our analyses (reducing the number of diseases associated with one or more genes to 3856). We next removed diseases classified as congenital, hereditary, and neonatal diseases and abnormalities (due to the lack of fetal data within the gene expression data set) and neoplasms (due to the previous observation that unlike other diseases, cancer-associated genes tend not to be overexpressed in the tissues in which the cancers are located [6]) using the disease classifications provided by DisGeNET (reducing the number of diseases to 2143). We then removed associations extracted from the literature supported only by a single evidence source (reducing the number of diseases to 1898). To increase the number of genes associated with each disease, we pooled the genes associated with sub-types of diseases wherever possible, by removing the number that follows the disease name in many DisGeNET entries (reducing the number of diseases to 1679). We also removed genes for which there were no gene expression data in the expression data set (reducing the number of diseases to 1557). Finally, we removed diseases that did not map to a disease medical subject heading (MeSH) term (from trees C and F03 of the 2015 MeSH tree structure, reducing the number of diseases to 1544). All genes were mapped to Ensembl gene identifiers using the biomaRt R package [24].

The GSC method works by measuring network distances between pairs of genes and therefore cannot be applied to diseases with only a single associated gene. For this reason, it is not possible to apply the GSC method to all of the 1544 diseases with one or more associated genes. We have demonstrated that the GSC method works well when applied to diseases with six or more associated genes (see ‘Parameter selection’ below). Of the 1544 diseases with one or more associated genes, 196 diseases have six or more associated genes. In the main analyses, we therefore used this subset of 196 diseases.

### The STRING database

PPI data were obtained from the STRING database (v9.1) [12]. The STRING database integrates experimentally verified PPIs with additional data sources, including genomic context, gene coexpression data and text-mined data. These data are used to produce confidence scores for the interactions. We included only experimentally verified PPIs with a confidence score greater than 0.8 within the cell-type-specific interactomes (see ‘Parameter selection’ below for justification). We mapped each *Homo sapiens* protein identifier to an Ensembl gene identifier [24]. The cell-type-specific interactomes each contain 32,275 interactions between 7332 proteins.

### Gene expression data

The FANTOM Consortium performed cap analysis of gene expression (CAGE) using single-molecule cDNA sequencing to identify transcription start sites (TSSs) and quantify their expression in *H. sapiens* and *Mus musculus* primary cell, tissue and cell line samples [8]. In our analyses, we use only the 362 *H. sapiens* primary cell samples organized into facets by Andersson et al. [9]. Individual TSSs were identified by the FANTOM Consortium using decomposition peak identification [8].

We downloaded the annotated CAGE peak counts mapped to *hg19* from the FANTOM5 website. Peaks located within 500 bp of the 5^′^ end of a gene transcript were assigned to that gene. Gene names were mapped to Ensembl gene identifiers [24]. Those peaks not assigned gene transcripts or for which no Ensembl gene identifier could be found were removed. For each gene, we summed the counts of each assigned peak to produce a single gene-wise expression value, as described in Sardar et al. [25]. These counts were normalized using the relative log expression method implemented within the edgeR R package [26] to produce values representing gene-wise tags per million.

We grouped FANTOM5 samples representing the same primary cell-type population using the sample names. Andersson et al. further organized these groups into broader facets, based upon cell function and morphology [9]. We therefore refer to the finer sample name-based groups as sub-facets. This produces a two-level hierarchy of sample groupings containing the facets and sub-facets (Additional file 2: Table S2).

Some of the facets defined by Andersson et al. [9] contain cell samples of different potency. For example, the mesenchymal cell facet contains both somatic amniotic membrane cells and pluripotent mesenchymal stem cells. It has previously been demonstrated that gene expression changes with cell differentiation [27]. Therefore, we split the mesenchymal cell facet into three facets (mesenchymal somatic cell, mesenchymal precursor cell and mesenchymal stem cell) and the monocyte facet into two facets (monocyte and cd14+ monocyte derived endothelial progenitor cell) and assigned sub-facets based upon the potency of the samples.

We conducted quality control to remove spurious samples. This was done by comparing samples corresponding to the same cell-type population and discarding samples with expression profiles that differ strongly from the other samples. First, the sub-facets containing only one sample were removed. Next, samples were normalized so that their gene-wise expression values summed to 1. The Jensen–Shannon distance (JSD) between each sample in each sub-facet was then computed [28], as described by Andersson et al. [9]. The JSD provides a measure of expression profile similarity. Complete linkage agglomerative hierarchical clustering was run using the JSD to cluster the samples. The resulting tree was cut at a height of 0.35 to split the samples into discrete clusters within each sub-facet. For each sub-facet, samples not contained within the largest cluster were removed. If no cluster contained more than one sample, all samples mapped to the sub-facet were removed. In total, 331 (91.4 %) of the 362 primary cell samples passed this quality control procedure. The 31 samples that did not pass this procedure were discarded and not used in the later analyses.

Many of the 331 samples that passed quality control correspond to the same cell type. The analyses conducted in this study require a single expression profile for each cell type and it was therefore necessary to combine these replicate samples. As previously explained, each sub-facet contains samples corresponding to a single cell type and these sub-facets are further organized into broader facets. However, the cell types found throughout the body are not represented equally within these facets and sub-facets. Cell types that are less accessible or more difficult to isolate from primary tissues, such as pancreatic cells and dendritic cells, are less well represented. This can be seen by comparing the number of sub-facets contained within each facet: the dendritic cell facet contains three sub-facets, while the vascular-associated smooth muscle cell facet contains ten sub-facets. If we were to combine samples using the sub-facet groupings then we would produce a disproportionately large number of vascular-associated smooth muscle cell expression profiles, which would potentially have a deleterious effect on the later analyses. Many of the sub-facets contained within these larger facets may represent similar cell types and therefore contain samples with similar gene expression profiles. Therefore, to produce a single expression profile for each cell type whilst avoiding the production of similar expression profiles as a result of uneven cell-type representation, we considered the similarity of the expression profiles of the samples within each facet.

The JSD was used to measure the similarity of the expression profiles of the samples in each facet. If the mean JSD was greater than 0.25, we combined samples by their sub-facet, producing multiple expression profiles under the names of the sub-facets. If the mean JSD was less than 0.25, we combined samples by their facet, producing a single expression profile under the name of the facet. A value of 0.25 was chosen after manual inspection of the functional-similarity of the sub-facets contained within each facet, along with the mean JSD between the samples within each facet. As previously mentioned, the vascular-associated smooth muscle cell facet contains ten sub-facets, many of which are functionally similar. Conversely, the mesenchymal stem cell facet contains seven sub-facets, representing stem cell populations that will form diverse cell and tissue types, including adipose tissue, hepatic cells and osteoblasts. We speculated that disruption to these diverse cell and tissue types may produce different phenotypes and therefore chose a value that would produce distinct expression profiles for each of these sub-facets. The mean JSD between the mesenchymal stem cell facet samples is 0.259 and we therefore chose a value of 0.25 to produce distinct expression profiles for the sub-facets within this facet, whilst producing a single expression profile for those facets containing functionally similar sub-facets, including the vascular-associated smooth muscle cell facet. The gene-wise expression values from different samples were combined by computing the mean tags per million value. Using this procedure, expression profiles for 74 cell types were derived from the 331 samples.

Many cell types contain a small number of highly expressed genes. For example, in reticulocytes, the expression of HBB is 176,857 times higher than the median expression value of the gene across all cell types. Using these raw expression values to construct context-specific interactomes would produce interactomes containing a small number of very high-weight edges. This would prohibit the use of the random walk with restart (RWR) method to measure distances between vertex pairs as these high-weight edges would come to dominate the movement of the walker. Therefore, we percentile-normalized the gene-wise expression values, which ensures that the scores range between 0 and 1. This is similar to the approach taken by Hu et al. [11]. For each gene in each cell type, we divided the expression value by the mean expression value of that gene across all cell types:

(1) $$ e'_{i,l} = \frac{k e_{i,l}}{ \sum_{j=1}^{k} e_{i,j}}   $$

where *e*_*i*,*l*_ is the expression value of gene *i* in cell type *l*, *ei*,*l*′ is the relative expression value and *k* is the number of cell types. For each cell type, these relative expression values were then transformed into percentile scores *x*, distributed uniformly between 0 and 1. A score of 1 indicates that a gene is the most overexpressed gene in that cell type, while a score of 0 indicates that it is the most underexpressed.

The gene expression profile of hepatocytes differed from the expression profiles of the other cell types. After division by the mean gene expression value across all cells types (Eq. 1), 364 hepatocyte genes had a relative expression value greater than 57 (within the top 0.1 % of values). The next highest cell type was mesenchymal somatic cells with 71 and the median number across all cell types was six. The application of alternative sample normalization methods, including the upper quartile and trimmed mean of *M*-value methods [26], failed to resolve this. We therefore did not use the hepatocyte expression profile in our analyses. This reduced the total number of cell-type profiles from 74 to 73.

### Creating context-specific interactomes

We created 73 cell-type-specific interactomes using an approach based on the edge re-weight method of Magger et al. [16]. Each edge in graph *G* is assigned a weight depending on the expression score of the interacting genes. Let *G*=(*V*,*E*), where *V* is a set of *n* vertices and *E*⊆*V*×*V* is a set of *m* undirected edges between pairs of vertices. *w*_*i*,*j*,*l*_=*x*_*i*,*l*_×*x*_*j*,*l*_, where *w*_*i*,*j*,*l*_ is the weight of the edge connecting vertex *i* and vertex *j* in the network created using the gene expression data from cell type *l* and *x*_*i*,*l*_ is the percentile-normalized expression score of gene *i* in cell type *l*. Larger values of *w* indicate a greater likelihood that an interaction takes place. Unlike the method of Magger et al. [16], we applied no cutoff to the gene expression data.

### Gene set overexpression

The percentile-normalized gene expression scores were used to quantify the significance of overexpression. Let *S* be the set of genes associated with a disease. The significance of the overexpression of gene set *S* in cell type *l* was measured using a permutation-based approach. To create *u* permuted expression profiles, we randomly reassigned, for each gene, the expression scores and the cell type. The mean expression score of *S* was then computed in the observed and permuted expression profiles. An empirical *P* value was produced by computing the proportion of permuted expression profiles in which the mean expression score was greater than the mean expression score in the observed profile. A minimum *P* value of 1/*u* was applied to ensure that no *P* values equaled 0. We used 10,000 permutations throughout our analyses. This is the GSO method.

### Gene set compactness

The compactness score provides a measure of how strongly a set of vertices interact in a graph. The compactness score of vertex set *S* on graph *G* is the mean distance between pairs of vertices in *S* on *G* [29], where |*S*| is the size of *S* and *d*^*G*^(*i*,*j*) is the distance between vertex *i* and vertex *j* in graph *G*. We used a ranked version of the RWR method [13] to measure distance. For each disease, *u* permuted interactomes were created using expression profiles permuted using the same approach as in the GSO method. The compactness score was computed for each of the observed and permuted interactomes. We define the compactness score (*C*) as:

(2) $$  C(S,G) = \frac{\sum_{i,j \in S} d^{G}(i,j)}{|S|^{2}}  $$

Empirical *P* values were produced by computing the proportion of permuted interactomes in which the compactness score of *S* was smaller than the compactness score of *S* in the observed interactome. A minimum *P* value of 1/*u* was applied to ensure that no *P* values equaled 0. We used 10,000 permutations throughout the analyses. This is the GSC method.

This use of the compactness score differs from previous uses. In Cornish et al. [30], the compactness score is used to measure the significance of gene set clustering in a single network, through the permutation of genes in the gene set. In the GSC method described here, the compactness score is used to compare gene set clustering across multiple networks, through the permutation of the data used to create the networks.

### Computing network distances using the random walk with restart method

The RWR method measures distances between vertex pairs in a graph. Unlike simpler methods, such as the shortest paths method, the RWR method incorporates the entire structure of the graph when measuring distances. It has been shown to be more effective than the shortest paths method in tasks such as disease gene prioritization [13].

To measure the distance from vertex *i* to vertex *j*, a random walker is started from vertex *i*. At each time step, the walker can either move to a vertex directly connected to its current vertex, or move back to its starting vertex with restart probability *r*. In an unweighted graph, the probability that the walker moves to each connected vertex is uniform. In a weighted graph, the probability distribution is based upon the weights of the edges, so that the walker is more likely to travel along an edge of high weight. As the number of time steps increases, the probability that the random walker will be located at each vertex converges to a steady state [31].

We used a method based on the iterative approach described by Köhler et al. [13] to compute the RWR distances. Let *A* be the column-normalized adjacency matrix of graph *G*, using edge weights *w*. *p* is a probability matrix with dimensions equal to *n*, the number of vertices in *G*. The element $p^{t}_{i,j}$ is the probability that a walker starting from vertex *i* is located at vertex *j* at time *t*. The initial probability matrix *p*^0^ is an identity matrix. Probabilities can be computed iteratively using:

(3) $$ p^{t+1} = (1 - r) A p^{t} + rp^{0}  $$

Iterations are conducted until the change in the probability matrix across time steps (*p*^*t*^ and *p*^*t*+1^, measured using the Manhattan distance) falls below a cutoff. To save computational time, we computed only the distances between the vertices in the vertex set *S* and all of the vertices in the graph. A restart probability *r* of 0.7 was used (see ‘Parameter selection’ below for justification), along with an iteration cutoff of |*S*|×10^−5^. For each vertex *i*, vertices are ranked by their probability, so that the vertex that the random walker is most likely to be located on is ranked first. These ranks are used as the distances between the vertices in Eq. 2.

### Text-mining of disease–cell-type associations

Text-mining of the PubMed database was also used to identify disease-associated cell types [32]. This was done by first mapping each cell type and disease to one or more MeSH terms. These MeSH terms were then used to query the PubMed database and identify diseases and cell types that were co-mentioned in articles more frequently than expected by chance.

We mapped every FANTOM5 project facet and sub-facet to one or more MeSH terms. For many facets and sub-facets, no single MeSH term from the MeSH Cells tree (A11) contained enough anatomical information to differentiate it from the other facets and sub-facets. Therefore, we mapped some facets and sub-facets to two MeSH terms: one representing the cell type from the MeSH Cells tree (A11) and one containing additional anatomical information from an alternative anatomical MeSH tree. In these cases, we used the overlap of the results for each term when querying the PubMed database.

The FANTOM Consortium provides an ontology (FF) to which they map each facet and sub-facet [8]. This ontology contains cross-mapping to the cell ontology (CL) [1]. Using these ontologies, each facet and sub-facet was mapped to a CL term. If the FF term of a facet or sub-facet was not cross-mapped to a CL term, then its ancestral FF terms in the FF ontology were considered. If multiple ancestors or CL terms were found, or if no CL term was found, the most representative CL term was selected manually. For each CL term, MeSH terms from the MeSH Cells tree (A11) were obtained by querying the MeSH database. If a MeSH term did not contain sufficient anatomical information to differentiate the facet or sub-facet from others, then we used the Uberon anatomical ontology [33], which is also cross-referenced in FF. Anatomical MeSH terms were obtained by querying the MeSH database with the Uberon term when not available in the Uberon ontology.

Each disease was mapped to a MeSH term through United Medical Language System (UMLS) terms [34]. Each disease within the DisGeNET database is associated with a UMLS term. We used the UMLS Metathesaurus to map each UMLS term to a disease MeSH term (from trees C and F03). Diseases associated with UMLS terms not present in the UMLS Metathesaurus were mapped to MeSH terms manually by querying the MeSH database with the UMLS term.

While we have attempted to map each cell type and disease to a unique MeSH term or pair of MeSH terms, the lack of specific terms in some areas of the MeSH database prevented us from doing this. Therefore, some diseases and cell types are mapped to the same terms. Also, due to the ontological structure of the MeSH database, some of the MeSH terms mapped to facets, sub-facets and diseases are either the ancestors or offspring of other MeSH terms. We do not consider these relationships when comparing the text-mined associations to the associations produced using the GSC and GSO methods.

Fisher’s exact test was used to measure the significance of observing the number of articles co-mentioning terms given the number of articles mentioning the terms individually [35]. *P* values were obtained from a contingency table containing the number of co-occurrences of the cell type and disease, the cell type without the disease, the disease without the cell type and the remaining number of articles within the corpus (the corpus is the total number of articles within the PubMed database). PubMed was queried using the Entrez Programming Utilities (eUtils) [36] on 23 April 2015.

### Parameter selection

As previously described, cell-type-specific interactomes were created using physical interactions from the STRING database with confidence scores greater than 0.8. Distances were measured across these interactomes using a restart probability *r*. We measured the effect of these two parameters on the GSC method by applying the GSC method to cell-type-specific interactomes created using confidence cutoff scores of 0.0, 0.2, 0.4, 0.6 and 0.8 and using restart probabilities of 0.1, 0.3, 0.5, 0.7 and 0.9. Diseases from the DisGeNET data set with at least two associated genes were used. Performance was measured by comparing the disease–cell-type associations identified using the GSC method to the associations identified using text-mining. By considering the text-mined associations as true positives, we are able to estimate the precision, recall and F1 score for the GSC method. As previously explained, text-mining is likely to identify a number of false positives and therefore these performance scores are likely to be under-estimated. However, they provide us with a method of comparing GSC method performance across the parameter space.

Applying the GSC method to interactomes created using different confidence score cutoffs had little effect on method performance (Additional file 3: Table S3). We therefore applied a confidence score cutoff of 0.8 to reduce the density of the network.

Using restart probabilities between 0.1 and 0.9 had little effect on method performance (Additional file 3: Table S3). A previous method that used the RWR method to identify gene–phenotype relationships successfully used a restart probability of 0.7 [37]. For these reasons, we ran the GSC method with a restart probability of 0.7.

To identify the number of disease-associated genes required by the GSC method, we applied the GSC method to sets of diseases with similar numbers of associated genes (Additional file 4: Table S4). Each disease set contains at least 20 diseases. To create the disease sets, diseases were first sorted by their respective number of associated genes. Diseases were added to the disease sets in this sorted order, starting from the disease with the greatest number of associated genes. Diseases were added to the same disease set until the number of diseases in the set reached 20. When this occurred, a new disease set was created and the disease added to this new disease set. Diseases with the same number of associated genes were always added to the same disease set. Cell-type-specific interactomes were created using a confidence cutoff score of 0.8 and random walks completed using a restart probability of 0.7. Text-mined disease–cell-type associations were used to measure the performance of the GSC method on each disease set. As previously explained, the GSC method cannot be applied to diseases with only a single associated gene, as the method works by measuring the network distances between pairs of genes. The overlap between the GSC and text-mined associations is significant for diseases with three or more associated genes. However, there is a large improvement in GSC method performance when the number of associated genes increases from five to six. For this reason, we completed the analyses using the 196 diseases with six or more associated genes.

### Constructing a diseasome using causal cell types

We produced a diseasome by connecting each disease to the four diseases with which it correlated most strongly with respect to the cell types identified as associated by the GSC method. For each disease pair, Pearson’s product-moment correlation coefficient was computed using the − log10 of the *P* values corrected using the Benjamini–Hochberg procedure for multiple testing [38]. We removed diseases with no cell-type associations passing a false discovery rate (FDR) of 10 %. We chose to connect each disease to a fixed number of other diseases, rather than applying a correlation cutoff, as this allows for the identification of the strongest correlations for each disease. Adding edges between all disease pairs that pass a correlation cutoff produces a diseasome in which some diseases have a large degree, while other diseases have no connections. A value of four was chosen as it produced a diseasome in which clusters of diseases of the same class could easily be identified. We have also created diseasomes by connecting each disease to the two (Additional file 5: Figure S1), three (Additional file 6: Figure S2) and five (Additional file 7: Figure S3) diseases with which they correlate most strongly. Clustering of diseases of the same class can also be seen in these alternative diseasomes.

Vertices in the diseasome are colored by the class of the disease. These disease classes were obtained from MeSH. The mapping of DisGeNET diseases to MeSH terms employed in the text-mining was used to map the diseases to the MeSH ontology. For each disease, ancestors at the second level of the MeSH ontology were identified. Many diseases have multiple ancestors at this level. When diseases mapped to multiple ancestral terms, we chose the ancestral terms occurring most frequently across all diseases to represent each disease, as this reduced the number of classes represented in the network, making the disease clusters easier to identify. Diseases belonging to classes represented in the diseasome fewer than eight times were combined within the other class. Edges connecting two diseases of the same disease class are also colored using the color of the disease class. Vertices in the diseasome are arranged using the Fruchterman and Reingold layout algorithm implemented in the igraph R package [39] with the default parameters.

### Enrichment of high-weight edges in the monocyte-specific psoriasis sub-network

**Fig. 1:**
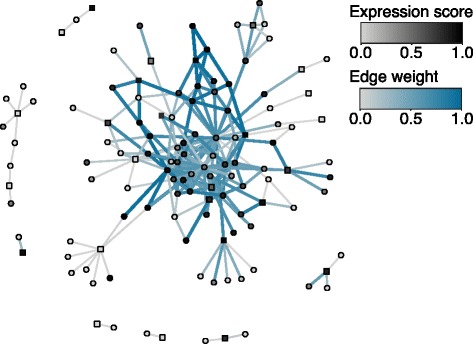
Monocyte-specific psoriasis sub-network. To create the sub-network, the protein products of all psoriasis-associated genes (squares) and their interacting partners (circles) were identified. To improve visual interpretation, psoriasis-associated genes with more than 15 interacting partners were removed. The more overexpressed each gene is in monocytes, the darker the color of the vertex. Edges with larger weights are darker blue and thicker. This sub-network is enriched with high-weight edges (*P*=0.0001 when genes with more than 15 interacting partners are not removed) that connect many of the disease-associated genes, suggesting that cell-type-specific interactomes may be useful in gene prioritization

The monocyte-specific psoriasis sub-network was created by identifying the protein products of genes associated with psoriasis and their interacting partners. In Fig. 1, proteins with more than 15 interacting partners were removed to improve visual interpretation. To determine whether the monocyte-specific psoriasis sub-network is enriched with high-weight edges, 10,000 permuted sub-networks were created by permuting the monocyte percentile-normalized gene expression scores. The number of edges with a weight greater than 0.90 (within the top 1 % of edge weights in the monocyte-specific interactome) were counted for the observed sub-network and each of the permuted sub-networks. Only 1/10,000 of the permuted sub-networks contained a greater number of high-weights edges than the observed sub-network, producing an empirical *P* value of 0.0001. This enrichment analysis was completed without removing proteins with more than 15 interacting partners.

## Results

### Using gene set compactness to identify disease-manifesting cell types

The FANTOM5 project used CAGE in different cell types to identify TSSs and quantify their expression [8, 9]. We combined the TSS-wise expression values to produce gene-wise percentile-normalized [25] relative expression scores (see ‘Methods’). In total, gene expression profiles were produced for 73 cell types.

Disease-associated genes are often enriched within certain pathways [2], the disruption of which leads to the disease. Cellular pathways are represented within PPI networks and because of this, sets of disease-associated genes tend to cluster within PPI networks [4, 40]. This is exemplified by the results produced by gene prioritization tools such as PRINCE [14], which use the clustering of disease-associated genes within PPI networks to prioritize candidate genes. Pathways whose disruption leads to a disease are likely to be active within the cell types associated with the disease. Therefore, we would expect disease-associated genes to cluster most strongly in the interactomes specific to the disease-manifesting cell types, providing us with a method of identifying these cell types. As no cell-type-specific interactomes are available, we integrated the FANTOM5 project cell-type-specific gene expression data with 32,275 PPIs from the STRING database [12] to build the largest collection of cell-type-specific interactomes currently available. In these interactomes, each vertex represents a gene and each edge a physical interaction weighted using the product of the gene pair’s percentile-normalized gene expression scores (Additional file 8: Figure S4).

**Fig. 2:**
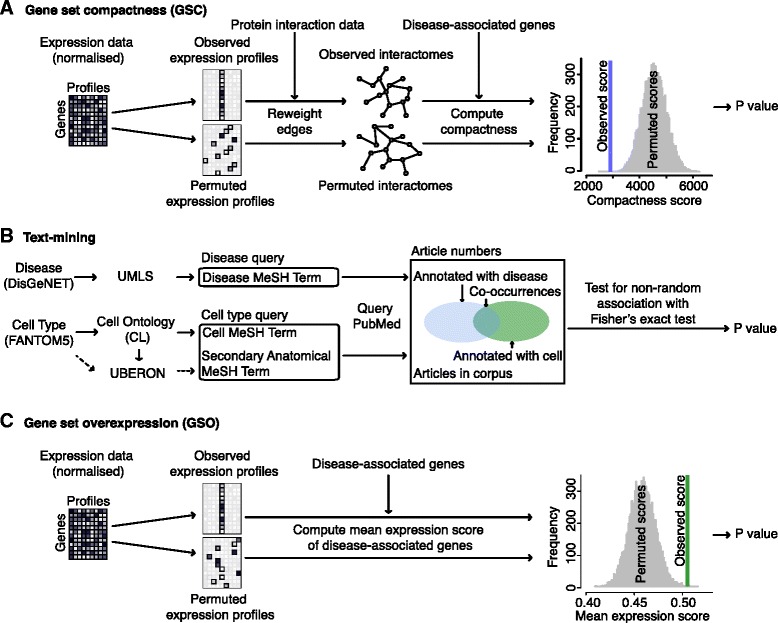
Overview of the GSC, text-mining and GSO methods. (**A**) For the GSC method, percentile-normalized relative gene expression scores are integrated with PPI data to create interactomes. Permuted interactomes are created by permuting the expression scores. The compactness score of the disease-associated gene set is computed for each observed and permuted interactome and empirical *P* values produced by counting the proportion of permuted compactness scores less than the observed compactness score. (**B**) To complete the text-mining, diseases from DisGeNET and cell types from the FANTOM5 project were mapped to MeSH terms using a number of controlled vocabularies. These MeSH terms were then used to query PubMed and count the number of articles individually and co-mentioning terms. Fisher’s exact test was used to determine whether the number of co-mentioning articles is greater than expected by chance. (**C**) For the GSO method, percentile-normalized gene expression scores are used to create observed and permuted expression profiles. The mean expression score of the disease-associated gene set is then computed for the observed and permuted expression profiles. Empirical *P* values are computed by counting the numbers of permuted scores greater that each observed score

We introduce the compactness score [29] to identify the cell-type-specific interactomes within which sets of disease-associated genes are significantly more clustered than expected by chance (Fig. 2A) and thereby identify disease-manifesting cell types. The compactness score is defined as the mean distance between pairs of vertices in a set in a graph. The smaller the compactness score of a vertex set, the stronger the interactions between the vertices in the set. If a vertex set interacts more strongly than expected by chance, then the vertex set can be said to cluster [30].

**Fig. 3:**
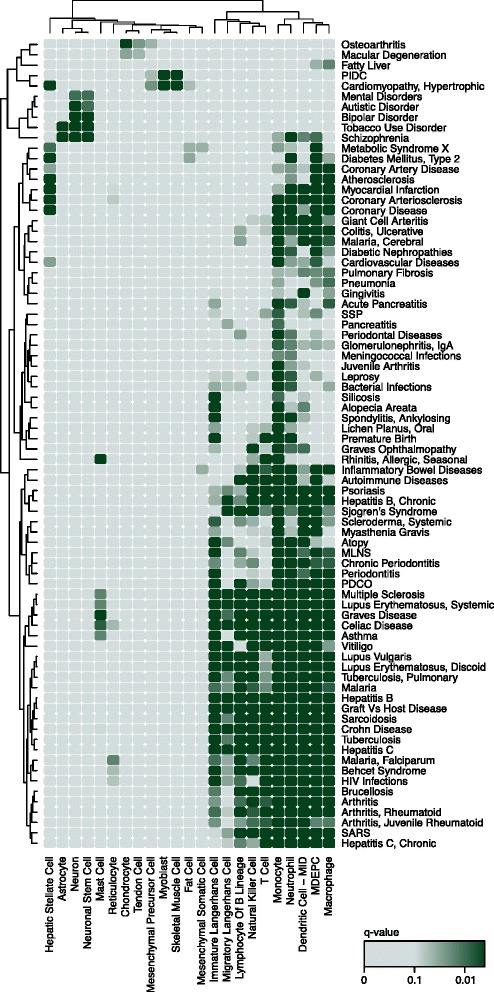
Heat map of a subset of the disease–cell-type associations identified by the GSC method. The darker the shade of green, the stronger the association. *P* values have been corrected using the Benjamini–Hochberg procedure for multiple testing. Each cell type and disease is involved in at least two associations with *q*<0.1. Cell types and diseases have been clustered using complete-linkage hierarchical clustering. Additional file 9: Figure S5 contains the complete set of associations identified. MDEPC: monocyte-derived endothelial progenitor cell, MLNS: mucocutaneous lymph node syndrome, MID: monocyte immature derived, PDCO: pulmonary disease, chronic obstructive, PIDC: primary idiopathic dilated cardiomyopathy, SARS: severe acute respiratory syndrome, SSP: subacute sclerosing panencephalitis

To quantify clustering significance, we permute the expression profiles and use these permuted profiles to build permuted interactomes (see ‘Methods’). A *P* value is produced for each cell type by comparing the compactness score of the vertex set on the observed and permuted cell-type-specific interactomes. In total, this method identified 660 associations between 73 cell types from the FANTOM5 project data and 196 diseases from the DisGeNET database of disease–gene associations [3] (Fig. 3, Additional file 9: Figure S5, Additional file 10: Table S5, Additional file 11: Table S6, at an FDR of 10 % computed using the Benjamini–Hochberg procedure for multiple testing [38]). This set of 196 diseases contains the diseases in the DisGeNET database associated with six or more genes, after the application of multiple filtering steps used to select the highest-quality associations between human diseases and genes. This is the GSC method.

### Using text-mining to identify disease-manifesting cell types

To provide an assessment of the validity of the above approach, we used text-mining to produce an independent literature-based association of diseases to cell types. Text-mining has previously been used to identify associations between diseases and various biological entities, including genes [5] and tissues [6]. However, to our knowledge, no study has used this approach to identify associations between diseases and a large number of cell types, possibly due to the difficulties in identifying articles that mention highly specific cell types.

We used the PubMed database of articles to identify associations between cell types and diseases (Fig. 2B). Articles within the PubMed database are annotated with MeSH terms, a controlled vocabulary that describes the topics of each article [36]. Whether two MeSH terms are associated or not can be determined by counting the number of articles that mention each term individually and comparing this to the number of articles that mention both terms. If the number of co-mentions is greater than expected given the number of individual mentions, then the terms can be said to be associated [32].

To conduct the text-mining, it was first necessary to map the cell types and diseases to MeSH terms (Additional file 12: Table S7 and Additional file 13: Table S8). This was done using the cross-referencing provided by the FANTOM Consortium and DisGeNET, a number of controlled vocabularies, including UMLS [34] and Uberon [33], the MeSH database and manual curation. Mapping diseases was relatively simple, as there exists a unique MeSH term for the majority of diseases in DisGeNET. Mapping cell types was more difficult, due to the relatively small number of cell types represented in the MeSH database. For example, the 73 cell types include five different types of fibroblast (fibroblasts of the choroid plexus, gingiva, lymphatic vessel, periodontium and tunica adventitia of artery). The MeSH database does not contain a unique term for each of these fibroblast sub-types. We therefore combined the most-representative cellular MeSH term (in this example ‘Fibroblast’) with anatomical MeSH terms to differentiate between the cell types. These combined MeSH terms were then used to query PubMed. A one-tailed Fisher’s exact test was used to identify cell-type/disease pairs co-mentioned more frequently than expected by chance [35]. In total, text-mining identified 1150 associations between the 73 cell types and the 196 diseases (Additional file 14: Table S9).

A disadvantage of this method is that it does not take into account the context in which a cell type or disease is mentioned in an article. This prevents us from distinguishing between those cell types directly affected by the disease-associated genes and those cell types indirectly affected later in the development of the disease. Psoriasis is a chronic skin condition generally considered to be caused by environmental and genetic factors and disruption to the immune system [41]. Skin cell types are strongly affected by psoriasis and they are therefore often mentioned in articles referring to the disease. While our text-mining identifies skin cells as being strongly associated with the disease (epidermal keratinocytes: *q*=10^−319^, the *q* value is analogous to the *P* value and represents the minimum FDR at which the association can be declared significant), it is unable to determine whether the disease-associated genes disrupt processes and pathways active within the skin cells or whether the skin cells are indirectly affected.

We used the text-mined disease–cell-type-associations to measure the performance of the GSC method when applied to diseases with different numbers of associated genes (Additional file 4: Table S4). Sets of disease–cell-type associations were created by applying an FDR cutoff of 10 % to the results. As the number of genes associated with each disease increases, the overlap between the GSC and text-mined associations also increases, indicating that the GSC method performs better on diseases with a greater number of associated genes. The overlap between the GSC and text-mined associations is significant for diseases with three or more associated genes. All of these associations are contained within the additional files (Additional file 10: Table S5 and Additional file 14: Table S9). There is a large increase in the significance of the overlap when the number of associated genes increases to six. For this reason, we decided to complete the remaining analyses using the 196 diseases in the DisGeNET data set associated with six or more genes.

### Comparison of disease-manifesting cell-type identification methods

Alongside the GSC and text-mining methods, we used the overexpression of sets of disease-associated genes to identify associated cell types (Fig. 2C, Additional file 15: Table S10 and Additional file 16: Table S11, see ‘Methods’), an approach previously found to be successful [11]. This is the GSO method.

Although the GSC and text-mining methods identify different associations (Additional file 17: Figure S6 and Additional file 18: Figure S7), the overlap is significant at FDRs of 5, 10 and 20 % (Table 1 and Additional file 19: Table S12), demonstrating that a significant proportion of the associations identified by the method are supported by published literature, providing validation for the method. At these FDRs, the overlap between the GSO and text-mining results is also significant. While a similar proportion of GSC- and GSO-identified associations are supported by text-mining (Additional file 20: Figure S8), the advantage of using cell-type-specific interactomes to explore the molecular mechanisms that drive disease is their ability to identify previously unidentified disease-associated genes and pathways. These results demonstrate that disease-associated genes interact more strongly in the interactomes of the disease-manifesting cell types. These interactomes are therefore more likely to be informative when identifying previously unidentified disease-associated genes.

**Table 1:** Number of disease–cell-type associations identified by the GSC and GSO methods supported by text-mining at a 10 % FDR. Text-mining overlap significance was computed using Fisher’s exact test

Method	GSC	GSO
Supported by text-mining	320	269
Not supported by text-mining	340	294
Text-mining overlap significance	5.80×10^−183^	1.57×10^−149^

The GSC and GSO methods identify different sets of associated cell types, indicating that they represent complementary approaches. However, there is still a large amount of support between the GSC and GSO methods (Additional file 21: Figure S9). For 64.0 % of diseases, at least 50 % of the associations identified by the GSC method are supported by the GSO method.

Using the MeSH term mapped to each disease and the cell ontology term mapped to each cell type [1], it is possible to identify classes of diseases and cell types. The GSC, GSO and text-mining methods identify different numbers of associations between diseases and cell types of different classes (Table 2). A greater proportion of the cell types identified by the GSC and GSO methods as being associated with immune system diseases are cell types of the immune system. Conversely, a greater proportion of the cell types identified by text-mining as being associated with cardiovascular diseases and mental disorders are cell types of the cardiovascular system and neural cell types, respectively. The GSC and GSO methods identify low numbers of cardiovascular cell types as being associated with cardiovascular diseases, compared to text-mining. This may indicate that these methods are less effective when applied to this disease class. Many of the cell types identified as associated with cardiovascular diseases by the GSC and GSO methods are cell types of the immune system, possibly reflecting an important role for the immune system in cardiovascular disease development [42]. As previously mentioned, the text-mining method used in this study does not take into account the context in which a cell type or disease is mentioned and therefore cannot distinguish between the cell types directly affected by the disease-associated genes and indirectly affected cell types. This may contribute to the differences seen between the methods. To test this, text-mining methods that are able to incorporate the contexts in which cell types and diseases are mentioned in articles will need to be developed and applied.

**Table 2:** Associations between diseases and cell types of particular classes. The table shows the proportion of cell types in a class identified as associated with a disease class

Disease class	Cell-type class	GSC	GSO	Text-mining
Cardiovascular diseases	Cardiovascular cells	4.7 % (5/107)	7.2 % (7/97)	27.3 % (50/183)
Cardiovascular diseases	Immune system cells	42.1 % (45/107)	36.1 % (35/97)	19.1 % (35/183)
Immune system diseases	Immune system cells	87.8 % (129/146)	86.5 % (109/126)	58.4 % (118/202)
Mental disorders	Neural cells	51.6 % (16/31)	38.7 % (12/31)	66.7 % (12/18)

The MeSH database and cell ontology were used to identify diseases and cell types belonging to each class. A disease and cell type were said to belong to a class if they were descendants of the following terms: cardiovascular diseases (C14), cardiovascular cells (CL:0002139 and CL:0002494), immune system diseases (C20), immune system cells (CL:0000738), mental disorders (F03) and neural cells (CL:0002319). An FDR cutoff of 10 % was applied to the results of each method to produce sets of disease–cell-type associations

### Examples of disease-manifesting cell types identified by gene set compactness

The GSC method identifies well-characterized associations between diseases and cell types, as well as associations that warrant further study. The integration of multiple data sources, such as cell-type-specific gene expression data, PPI data and disease-associated genes, provides the key to identifying the cellular basis of disease.

A large number of associations are identified between cell types of the immune system and autoimmune disorders, such as rheumatoid arthritis and macrophages (*q*=0.005). In addition, susceptibilities to infectious diseases, including malaria falciparum and hepatitis B, are associated with immune system cell types, including monocytes and neutrophils (all *q*=0.005). Neurons and neuronal stem cells are identified as being associated with a number of mental disorders, including psychotic disorders such as schizophrenia and bipolar disorder, and substance abuse disorders such as tobacco use disorder and alcoholism (all *q*=0.005). The method also identifies associations between highly localized diseases and known associated cell types, including lens epithelial cells and retinal diseases (*q*=0.005).

The GSC method identifies associations between mast cells and a number of diseases, including asthma and multiple sclerosis (MS, both *q*=0.017). While the involvement of mast cells in allergic diseases such as asthma is well understood [43], the association between mast cells and MS is less well characterized. Although text-mining does not identify an association between mast cells and MS, there is some evidence that mast cells may play a role in the initiation and progression of the disease. Mast cells are known to be key regulators of the permeability of the blood–brain barrier [44] and decreased permeability of this barrier is one of the earliest signs of MS [45]. Furthermore masitinib, an inhibitor of mast cell activity, migration and survival, was seen to produce small but non-significant improvements in MS patients in a phase 2a clinical trial [46]. A phase 2b/3 clinical trial of masitinib and MS is currently underway (ClinicalTrials.gov ID: NCT01433497).

Preeclampsia is defined as the new onset of proteinuria and hypertension during the second half of pregnancy and affects 5–8 % of pregnancies [47]. Endothelial cells, immune cells and adipocytes have all been implicated in the development of preeclampsia. Endothelial cells are essential in the remodeling of maternal vessels to provide oxygen and nutrients to the developing fetus and placenta [48] and aberrant formation of these endothelial cells prevents these changes from occurring [49]. It has been suggested that natural killer cells may contribute to this endothelial cell dysfunction through the production of signaling proteins that affect the migration of the endothelial cells [50]. Adipocytes produce adipokines known to affect endothelial cell function and have therefore also been implicated in endothelial cell dysfunction [51]. Furthermore, obesity is known to both affect the production of these adipokines and increase the risk of preeclampsia threefold [51]. The GSC method identifies fat cells as the cell type most strongly associated with preeclampsia (*q*=0.059) supporting the hypothesis that adipocytes influence the development of the disease, possibly through the aberrant production of adipokines.

Osteoarthritis is considered an age-related disease and affects 14 % of people over the age of 60 [52]. While many individuals exhibit age-related changes within their joints, only some display the symptoms associated with osteoarthritis [53]. Multiple genes have been identified as being associated with osteoarthritis, but how these genes influence the development of the disease is still not known. Chondrocytes are the only cell type residing in the adult cartilage matrix and are responsible for the repair of the cartilage [54]. Osteoarthritis-associated genes may therefore influence the development of the disease through the disruption of the chondrocytes and this repair process. Parts of the inflammatory complement system have been observed to be present at elevated levels in the synovial fluids of individuals with early-stage osteoarthritis [55]. Mice models of osteoarthritis genetically deficient in these complement components exhibit less cartilage loss than mice that are not deficient in these components [55]. This demonstrates the importance of the immune system in the development of osteoarthritis and raises the possibility that osteoarthritis-associated genes promote the development of the disease through the disruption of the cells of the immune system. However, the GSC method identifies chondrocytes as the cell type most significantly associated with osteoarthritis (*q*=0.005), supporting the hypothesis that chondrocyte dysregulation is key to the development of the disease. Inflammation may occur as a result of this dysregulation.

### Cell-type-based diseasome

**Fig. 4:**
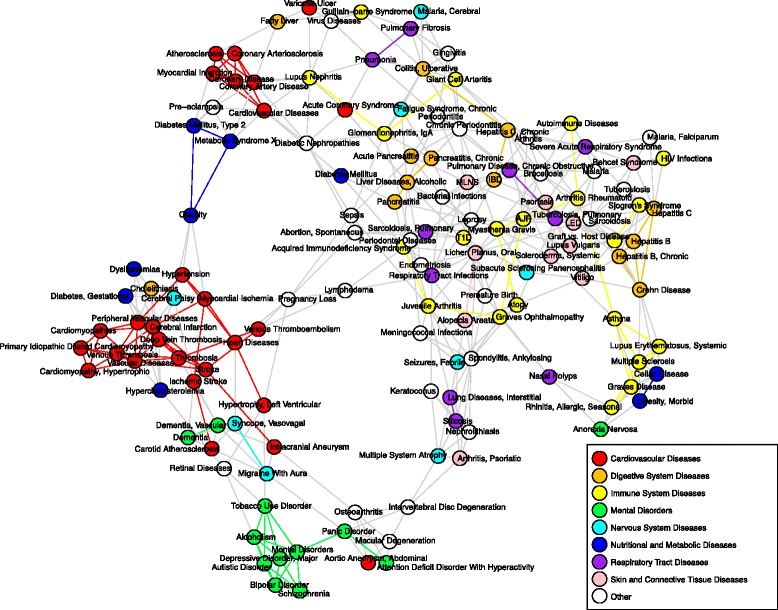
The disease-manifesting cell-type-based diseasome. Each vertex represents a disease and is colored by the disease class. Each disease is connected to the four diseases with which it correlates most strongly (Pearson’s product-moment correlation coefficient) with respect to the associated cell types identified by the GSC method. Edges connecting two diseases of the same class are colored with the class color. AJR: arthritis, juvenile rheumatoid, IBD: inflammatory bowel disease, LED: lupus erythematosus, discoid, MLNS: mucocutaneous lymph node syndrome, T1D: diabetes mellitus, type 1

A diseasome represents a network of diseases, connected by aspects of their etiology or treatment [4, 56]. It has been demonstrated that diseases that share molecular mechanisms are more likely to exhibit clinical co-morbidity and share drug treatments [56]. There is therefore much interest in mapping diseasomes, to aid in both disease study and drug re-purposing. Diseasomes have been created using shared associated genes [4], affected cellular pathways [57], co-occurrence in clinical records [58], common symptoms [59] and through the integration of these data [56]. We used the associated cell types identified by the GSC method to construct a diseasome based on common associated cell types (Fig. 4), the first of its kind.

Many diseases within the cell-type-based diseasome interact with diseases of the same class, an attribute shared with previously created diseasomes [4, 56]. Some diseases also interact with diseases of a different class, although many of these cases can be explained. For example, alopecia areata and systemic scleroderma are classified as skin and connective tissue diseases and interact strongly with immune system diseases, reflecting their autoimmune origin [60, 61].

The cell-type-based diseasome is also likely to be important in identifying novel associations between diseases and providing support for previously suggested associations. In the diseasome, obesity (classified as a body mass index greater than 30 [62]) is connected to a number of diseases with which it has an observed co-morbidity, including type II diabetes and hypertension [63]. However, morbid obesity (classified as a body mass index greater than 40 [62]) is most strongly correlated with four diseases of the immune system (three from the immune system diseases class and celiac disease). Increased macrophage numbers have been observed in the adipose tissues of both obese mice and obese humans and to contribute to the activation of inflammatory pathways in these tissues [64]. Group 2 innate lymphoid cells (ILC2s), a cell population involved in the regulation of adaptive immunity [65], have been recently observed to be critically involved in the regulation of brown and beige adipocytes [66], which are linked to the prevention of weight gain [67]. Furthermore, ILC2s have been observed at lower frequencies in the adipose tissues of obese humans compared to non-obese humans [66], leading to the suggestion that disruption of this immune system cell population may promote obesity in humans. These results, along with the connections in the diseasome between morbid obesity and diseases of the immune system, provide support for involvement of the immune system in the development and progression of severe forms of obesity. Integration of disease-manifesting cell-type data with additional genomic and clinical data will likely improve our ability to identify important connections between diseases.

## Discussion

A comprehensive understanding of genotype, gene expression and phenotype represents one of the main challenges of modern genetics. Studies looking at the *cis*-effect on gene expression could revolutionize our understanding of disease, but they require biological samples, which require invasive procedures such as biopsies. Moreover, the power of a study aimed at detecting the *cis*-effect on gene expression using biological samples is greatly limited by the presence of a non-homogeneous cell population in these tissues, such as blood, adipose or liver tissues. We have developed a novel approach that integrates cell-type-specific gene expression data with PPI data to identify those cell types through which sets of disease-associated genes exert their effect. Available for download is an R package called DiseaseCellTypes [22], containing implementations of the GSC and GSO methods and methods for building cell-type-specific interactomes.

It has previously been demonstrated that gene expression and PPI data can be used to identify the tissues in which diseases are manifested [16]. However, the lack of high-quality cell-type-specific expression data and the absence of a systematic map between cell types and diseases have limited research into disease-manifesting cell types. We have used text-mining to demonstrate that by comparing the clustering of disease-associated genes across cell-type-specific interactomes, it is possible to identify disease-manifesting cell types. It has recently been shown that the enrichment of disease-causing SNPs within cell-type-specific *cis*-regulatory regions can also be used to identify the cell types in which a disease is manifested [68]. New methods will need to be developed to integrate these and other data types to better identify cell types that underlie disease conditions.

The interactomes created in this study represent the largest collection of *H. sapiens* cell-type-specific interactomes created to date. As well as identifying disease-manifesting cell types, these cell-type-specific interactomes are also likely to be useful in the prioritization of disease genes and variants. Many gene-prioritization tools use PPI networks to identify genes whose protein products interact with the products of known disease-associated genes, under the hypothesis that these genes may be involved in the same functions and pathways [13, 14]. Figure 1 shows part of the monocyte-specific interactome, with genes known to be associated with psoriasis represented as squares. Many of the psoriasis-associated genes form part of a monocyte-specific sub-network enriched with high-weight edges (*P*=0.0001, see ‘Methods’). This sub-network could be used to prioritize additional psoriasis-associated genes. Genes that do not form part of this monocyte-specific sub-network may influence the disease through another cell type. While gene prioritization is beyond the scope of this study, the findings here indicate that a suitable prediction algorithm using cell-type-specific interactomes may aid in the prioritization of candidate genes.

While the GSC method is able to identify cell types associated with the majority of the 196 analyzed diseases, it identifies no associated cell type for 57 diseases (FDR of 10 %). Some of these cases may be due to the lack of the true disease-manifesting cell type within the tested expression profiles. Easily accessible cell types, such as those found circulating in the blood, are well represented in the FANTOM5 project primary cell samples. Cell types that are more difficult to extract (such as pancreatic cells) are less well represented and this prevents the identification of some cell types associated with some diseases (such as susceptibility to chronic pancreatitis).

All of the FANTOM5 primary cell samples represent healthy cells (rather than cancerous cell lines). It is known that the gene expression profiles of healthy cells differ significantly from those of diseased cells [69], representing a different set of active processes and pathways. This lack of diseased cell expression data may therefore further limit our ability to identify disease-manifesting cell types. As projects like FANTOM5 continue to grow, gene expression data from more conditions, such as disease states and developmental stages, will become available.

The lack of a single cell type underlying the development of a disease may prevent the identification of cell types associated with the disease. Primary ovarian insufficiency is defined as the failure of ovarian function before the age of 40 [70]. There are a large number of causes of this disorder, including hormonal dysfunction, autoimmunity and abnormal development of the ovaries [70]. The GSC method identifies no cell types as being associated with primary ovarian insufficiency, possibly due to the multifactorial nature of the disorder. Furthermore, the spread of genetic variants with small effect sizes across multiple genes may prevent the identification of disease-manifesting cell types.

The number of genes associated with each disease limits the effectiveness of the GSC method. However, as genome sequencing costs continue to decline, the number of identified disease-associated genes will increase, allowing the GSC method to be applied to new diseases. For many of the diseases in this analysis, the cell types directly affected by the associated genes have yet to be identified or are still being debated. Through the examples we provide, we demonstrate that the GSC method is able to provide information about these cell types.

## Conclusions

The data set produced in this study represents the first large-scale mapping of diseases to the cell types in which they are manifested. Our method successfully identifies many disease-associated cell types supported by previously published literature, as well as highlighting associations worthy of further investigation, including associations involving MS and preeclampsia. These associations will be useful in many tasks within disease research, such as prioritizing genetic variants and producing hypotheses about disease initiation and development. Furthermore, the cell-type-specific interactomes we have produced will be useful in analyzing the complex etiology of disease.

As the amount of cell-type-specific data increases, tools for identifying disease-manifesting cell types will become increasingly important. Increased availability of gene expression, proteomic and epigenetic data from additional cell types, developmental stages and disease states will facilitate the fine mapping of disease-manifesting cell types. These cell types will represent both candidates for further study and targets for therapeutics.

## Additional files

Additional file 1
**Table S1.** Disease–gene associations extracted from DisGeNET used in the analyses. DISEASE: Name of the disease in the analysis. GENE: Ensembl gene identifier of the associated gene. PUBMED_ID: PubMed identifiers reported by DisGeNET as supporting the association between the gene and the disease. SNP_ID: dbSNP identifiers reported by DisGeNET as linking the gene to the disease. (XLS 830 kb)

Additional file 2
**Table S2.** Mappings from each of the FANTOM5 project primary cell-type samples to its facet, sub-facet and cell-type group. CELL_TYPE: Name of the cell-type group that each sample was finally assigned to. The sample expression data was combined using these groups to produce expression profiles for 73 cell types. If the value in CELL_TYPE is NA then the sample was discarded, either due to a lack of replicate samples or because of the quality-control procedures. (XLS 42.5 kb)

Additional file 3
**Table S3.** Performance of the GSC method when applied to interactomes created using various confidence score cutoffs and run using various restart probabilities. All diseases with two or more associated genes were used. Performance is measured using associations identified by the text-mining as true positives. Sets of disease–cell-type associations were produced by applying an FDR cutoff of 10 % to the GSC and text-mining results. (XLS 10 kb)

Additional file 4
**Table S4.** Performance of the GSC method when applied to sets of diseases with different numbers of associated genes. Performance is measured using associations identified by the text-mining as true positives. Sets of disease–cell-type associations were produced by applying an FDR cutoff of 10 % to the GSC and text-mined associations. Overlap significance was measured using Fisher’s exact test. The GSC method cannot be applied to diseases with only one associated gene and for this reason, the corresponding statistics are not available. (XLS 7.50 kb)

Additional file 5
**Figure S1.** Disease-manifesting cell-type-based diseasome. Created by connecting each disease to the two diseases with which it correlates most strongly with respect to the associated cell types. (PDF 951 kb)

Additional file 6
**Figure S2.** Disease-manifesting cell-type-based diseasome. Created by connecting each disease to the three diseases with which it correlates most strongly with respect to the associated cell types. (PDF 980 kb)

Additional file 7
**Figure S3.** Disease-manifesting cell-type-based diseasome. Created by connecting each disease to the five diseases with which it correlates most strongly with respect to the associated cell types. (PDF 1090 kb)

Additional file 8
**Figure S4.** Example of the construction of a toy cell-type-specific interactome using gene expression and PPI data. Simulated (**A**) percentile-normalized gene expression scores and (**B**) PPI data are used. Edge weights (*w*) in the re-weighted network (**C**) are the product of the expression scores of the interactors. Higher weights are indicative of stronger interactions and therefore smaller distances between the interactors. To illustrate this, the distance along each edge is set as the reciprocal of the weight. The thickness and color of each edge is also proportional to the weight, with higher-weight edges represented by thicker and darker lines. Each vertex is colored by its gene expression score, so that vertices with higher expression scores are darker in color. From this toy example, it is clear that re-weighting the edges of the network results in the high-scoring vertices interacting more strongly (for example, *g* and *h*). (PDF 679 kb)

Additional file 9
**Figure S5.** Heat map of disease–cell-type associations identified by the GSC method between 73 cell types and 196 diseases. The darker the shade of green, the stronger the association. *P* values have been corrected for multiple testing using the Benjamini–Hochberg procedure. Cell types and diseases have been clustered using complete-linkage hierarchical clustering and reordered accordingly. (PDF 2960 kb)

Additional file 10
**Table S5.** Disease–cell-type association *P* values computed using the GSC method. (XLS 686 kb)

Additional file 11
**Table S6.** Disease-associated cell types identified by the GSC method with an FDR of 10 %. DISEASE: Disease name. CELL_TYPE: Cell-type name. P_VALUE: Unadjusted *P* value computed using the GSC method. Q_VALUE: Minimum FDR at which the association can be declared significant, computed using the Benjamini–Hochberg procedure for multiple testing. SIG_GSO: Whether the association is declared significant using the GSO method at an FDR of 10 %. SIG_TEXT: Whether the association is declared significant using text-mining at an FDR of 10 %. (XLS 78 kb)

Additional file 12
**Table S7.** Term mappings for the cell types used in the analyses. NAME: Cell-type name used throughout the analyses (derived from the FANTOM5 project sample name). CL_ID: Cell ontology identifier mapped to the cell type. MESH: The one or two MeSH terms mapped to the cell type used to conduct the text-mining. (XLS 14 kb)

Additional file 13
**Table S8.** Term mappings for the diseases used in the analyses. NAME: Disease name used throughout the analyses (obtained from DisGeN ET). UMLS: UMLS identifiers mapped to the disease. MESH: MeSH term mapped to the disease used to conduct the text-mining. (XLS 66 kb)

Additional file 14
**Table S9.** Disease–cell-type association *P* values computed using the text-mining method. (XLS 438 kb)

Additional file 15
**Table S10.** Disease–cell-type association *P* values computed using the GSO method. (XLS 686 kb)

Additional file 16
**Table S11.** Disease-associated cell types identified by the GSO method with an FDR of 10 %. DISEASE: Disease name. CELL_TYPE: Cell-type name. P_VALUE: Unadjusted *P* value computed using the GSO method. Q_VALUE: Minimum FDR at which the association can be declared significant, computed using the Benjamini–Hochberg procedure for multiple testing. SIG_GSC: Whether the association is declared significant using the GSC method at an FDR of 10 %. SIG_TEXT: Whether the association is declared significant using text-mining at an FDR of 10 %. (XLS 69 kb)

Additional file 17
**Figure S6.** Heat map comparing the disease–cell-type associations identified by the GSC method and text-mining. Differences are compared by first correcting the *P* values for multiple testing using the Benjamini–Hochberg procedure. The −log10 of the text-mined *q* value is then subtracted from the −log10 of the GSC method-computed *q* value. If the GSC method identifies the association as more significant, the corresponding cell is colored red. If text-mining identifies the association as more significant, the cell is colored blue. If the significance level is similar between methods, the cell is colored white. (PDF 3190 kb)

Additional file 18
**Figure S7.** Venn diagrams of the disease–cell-type associations **(A)** supported and **(B)** not supported by text-mining that are also identified by the GSC and GSO methods. Sets of associations were produced by applying an FDR cutoff of 10 % to the GSC, GSO and text-mining results. (PDF 677 kb)

Additional file 19
**Table S12.** The number of disease–cell-type associations identified by the GSC and GSO methods supported by text-mining at FDRs of 5, 10 and 20 %. Precision, recall and the F1 score are computed using associations identified by the text-mining method as true positives. Overlap significance was measured using Fisher’s exact test. (XLS 7.50 kb)

Additional file 20
**Figure S8.** The proportion of disease–cell-type associations identified by the GSC and GSO methods supported by text-mining at various cutoffs. Disease–cell-type associations were ranked by their *q* value. The number of associations represents the size of the set of the top-ranked disease–cell-type associations. To compute the proportion of disease–cell-type associations supported by text-mining, it was necessary to apply an FDR cutoff to the text-mining results. Here we use three cutoffs: **(A)** 10 %, **(B)** 1 % and **(C)** 0.1 %. The GSC method assigned 219 associations the lowest-possible *P* value, while the GSO method assigned 211 associations this *P* value. It was not possible to order these top-ranked associations and therefore the GSC and GSO curves start at 219 and 211 respectively. (PDF 706 kb)

Additional file 21
**Figure S9.** Histograms showing the proportions of disease-associated cell types identified by one method supported by another. An FDR cutoff of 10 % was applied to the results of each method to produce sets of disease–cell-type associations. For each disease, the proportion of associations identified by method *m*_1_ supported by method *m*_2_ was computed by dividing the number of associations identified by method *m*_1_ by the number of associations identified by both method *m*_1_ and *m*_2_. A value of 1 indicates that all associations identified by method *m*_1_ are supported by method *m*_2_ and a value of 0 indicates that no associations identified by method *m*_1_ are supported by method *m*_2_. Diseases where both methods identified no associated cell types were removed. There is a large amount of support between the GSC and GSO methods. For 64.0 % of diseases, at least 50.0 % of associations identified by the GSC method are supported by the GSO method. Similarly, for 64.8 % of diseases, at least 50.0 % of associations identified by the GSO method are supported by the GSC method. (PDF 1050 kb)

## Abbreviations

CAGE: cap analysis of gene expression
FDR: false discovery rate
GSC: gene set compactness
GSO: gene set overexpression
JSD: Jensen–
Shannon distance; MeSH: medical subject heading
MS: multiple sclerosis
PPI: protein–
protein interaction; RWR: random walk with restart
TSS: transcription start site
UMLS: United Medical Language System
