# Supplementary figures and images for: Exploring the cellular basis of human disease through a large-scale mapping of deleterious genes to cell types
Source: Genome Med. 2015 Sep 1;7(1):95. doi: 10.1186/s13073-015-0212-9 (PMC4557825; doi:10.1186/s13073-015-0212-9)

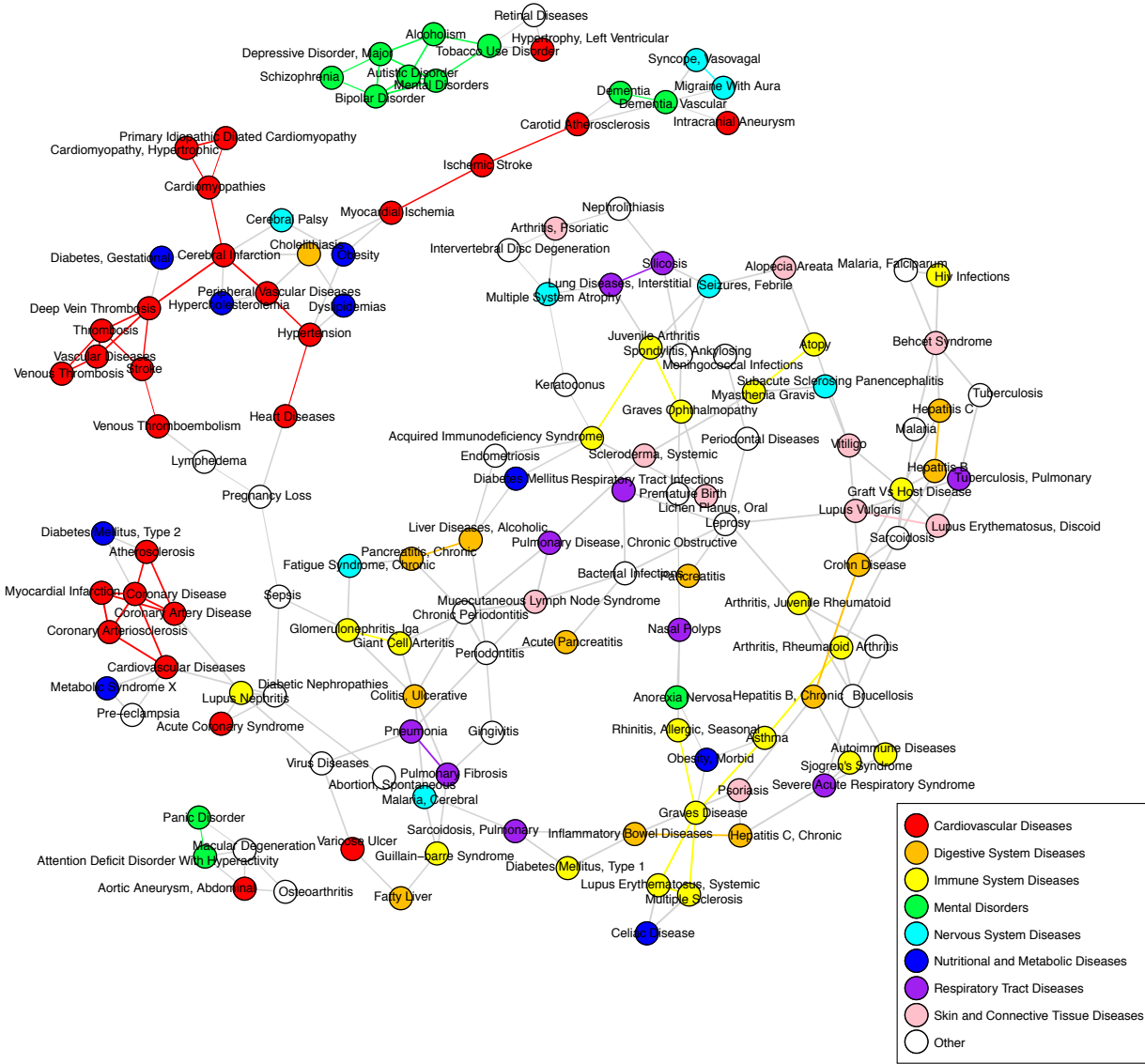

Supplement: Additional file 5 — Figure S1. Disease-manifesting cell-type-based diseasome. Created by connecting each disease to the two diseases with which it correlates most strongly with respect to the associated cell types. (PDF 951 kb) [file 13073_2015_212_MOESM5_ESM.pdf]

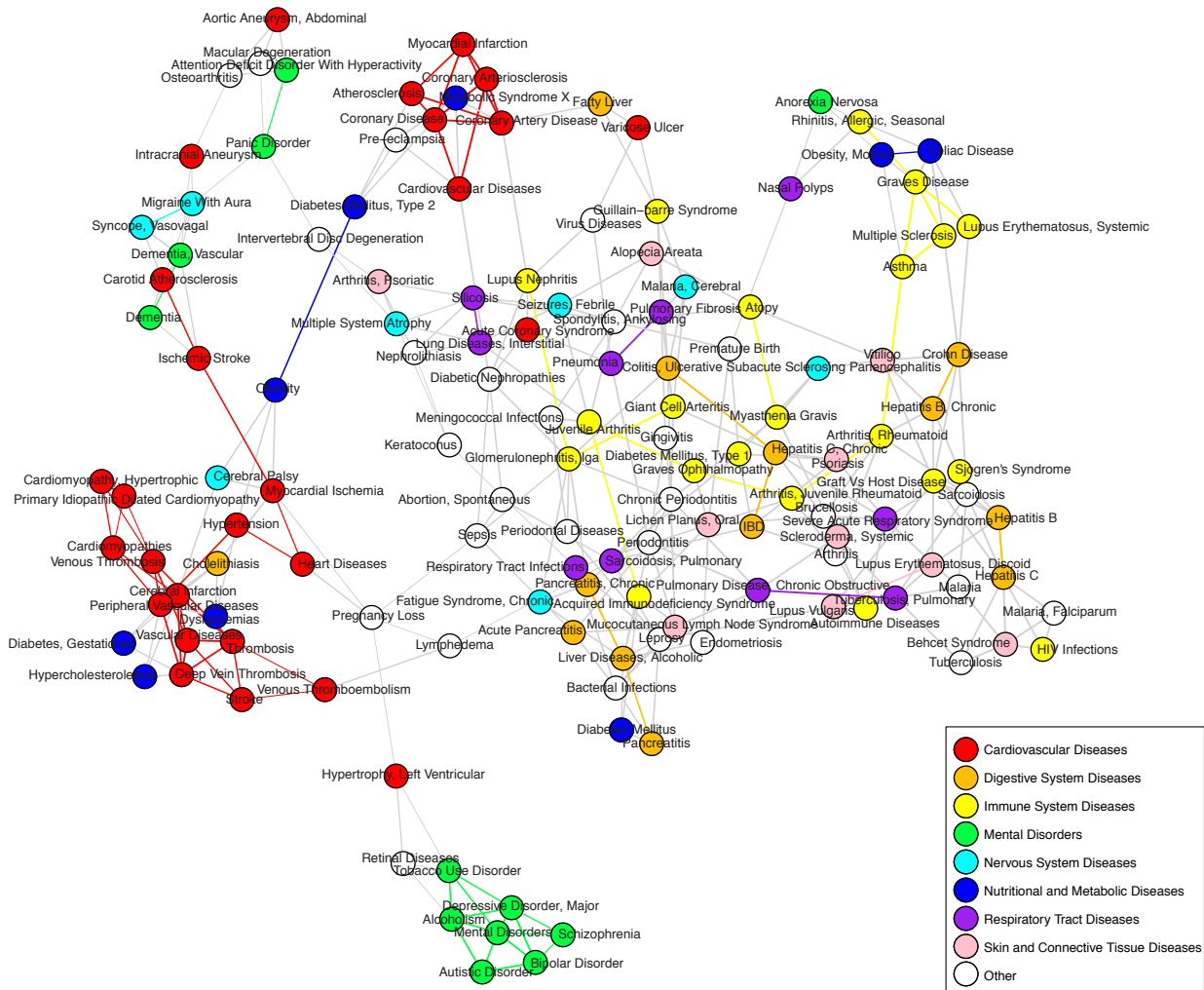

Supplement: Additional file 6 — Figure S2. Disease-manifesting cell-type-based diseasome. Created by connecting each disease to the three diseases with which it correlates most strongly with respect to the associated cell types. (PDF 980 kb) [file 13073_2015_212_MOESM6_ESM.pdf]

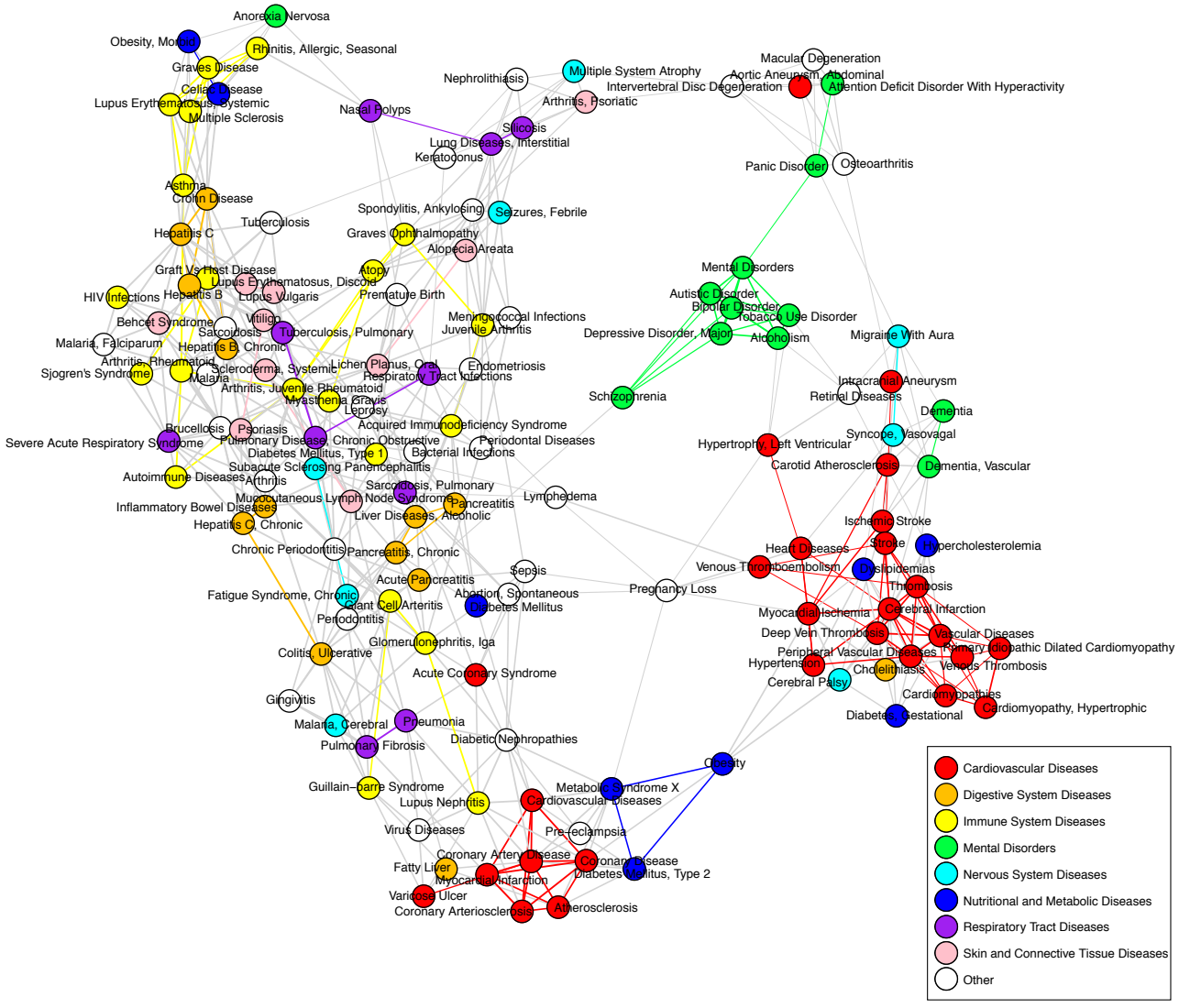

Supplement: Additional file 7 — Figure S3. Disease-manifesting cell-type-based diseasome. Created by connecting each disease to the five diseases with which it correlates most strongly with respect to the associated cell types. (PDF 1090 kb) [file 13073_2015_212_MOESM7_ESM.pdf]

**A) Expression data**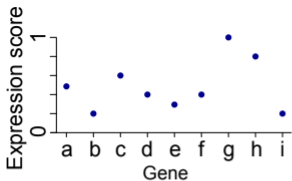**B) Protein-protein interaction data**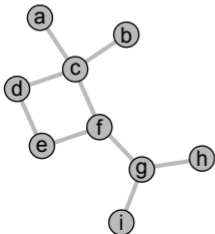**C) Edge-reweighted network**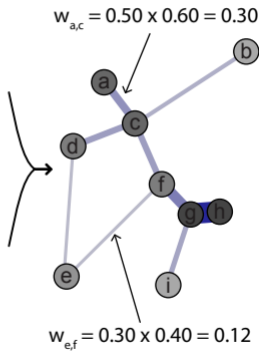

Supplement: Additional file 8 — Figure S4. Example of the construction of a toy cell-type-specific interactome using gene expression and PPI data. Simulated (A) percentile-normalized gene expression scores and (B) PPI data are used. Edge weights (w) in the re-weighted network (C) are the product of the expression scores of the interactors. Higher weights are indicative of stronger interactions and therefore smaller distances between the interactors. To illustrate this, the distance along each edge is set as the reciprocal of the weight. The thickness and color of each edge is also proportional to the weight, with higher-weight edges represented by thicker and darker lines. Each vertex is colored by its gene expression score, so that vertices with higher expression scores are darker in color. From this toy example, it is clear that re-weighting the edges of the network results in the high-scoring vertices interacting more strongly (for example, g and h). (PDF 679 kb) [file 13073_2015_212_MOESM8_ESM.pdf]

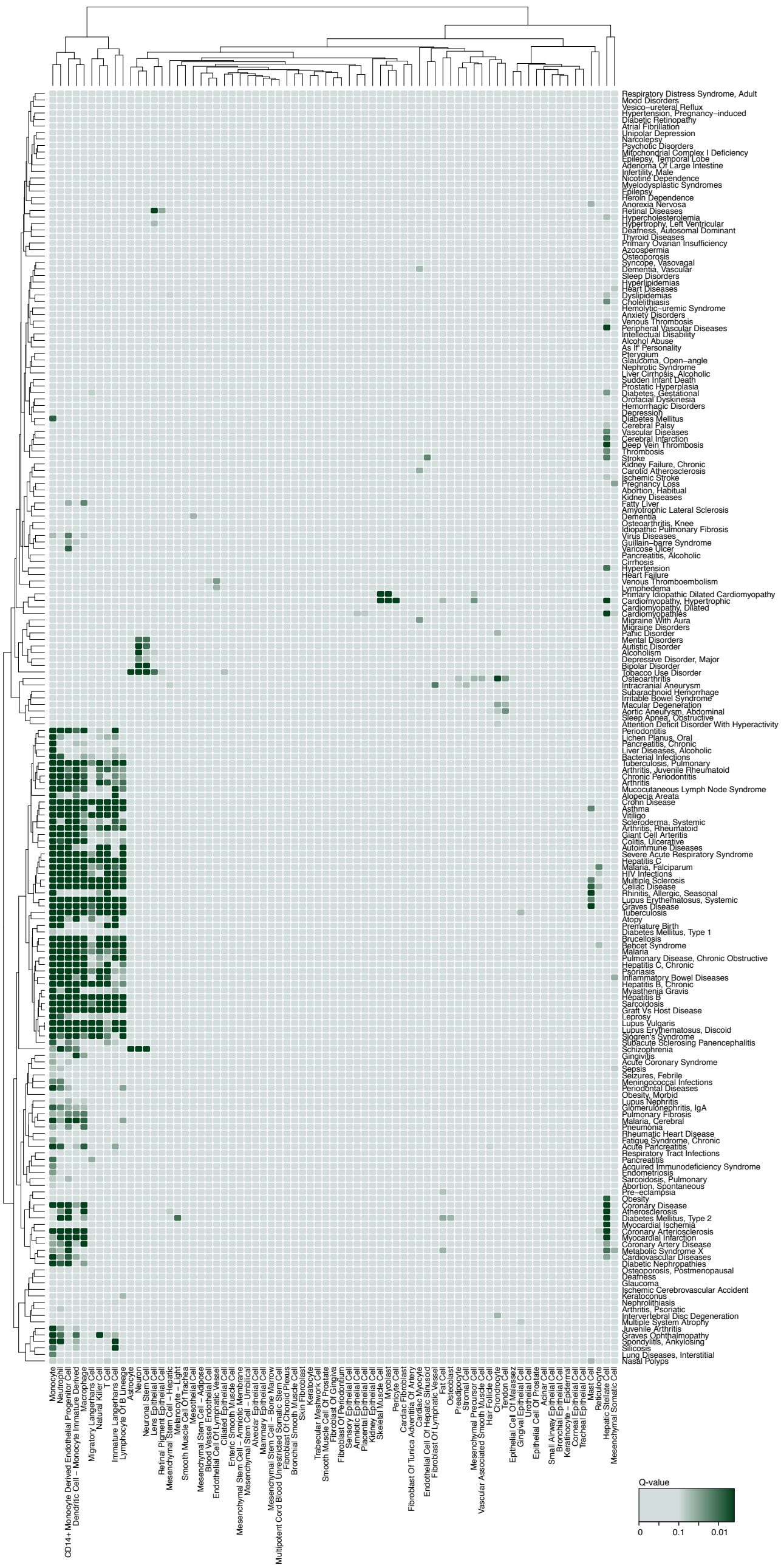

Supplement: Additional file 9 — Figure S5. Heat map of disease–cell-type associations identified by the GSC method between 73 cell types and 196 diseases. The darker the shade of green, the stronger the association. P values have been corrected for multiple testing using the Benjamini–Hochberg procedure. Cell types and diseases have been clustered using complete-linkage hierarchical clustering and reordered accordingly. (PDF 2960 kb) [file 13073_2015_212_MOESM9_ESM.pdf]

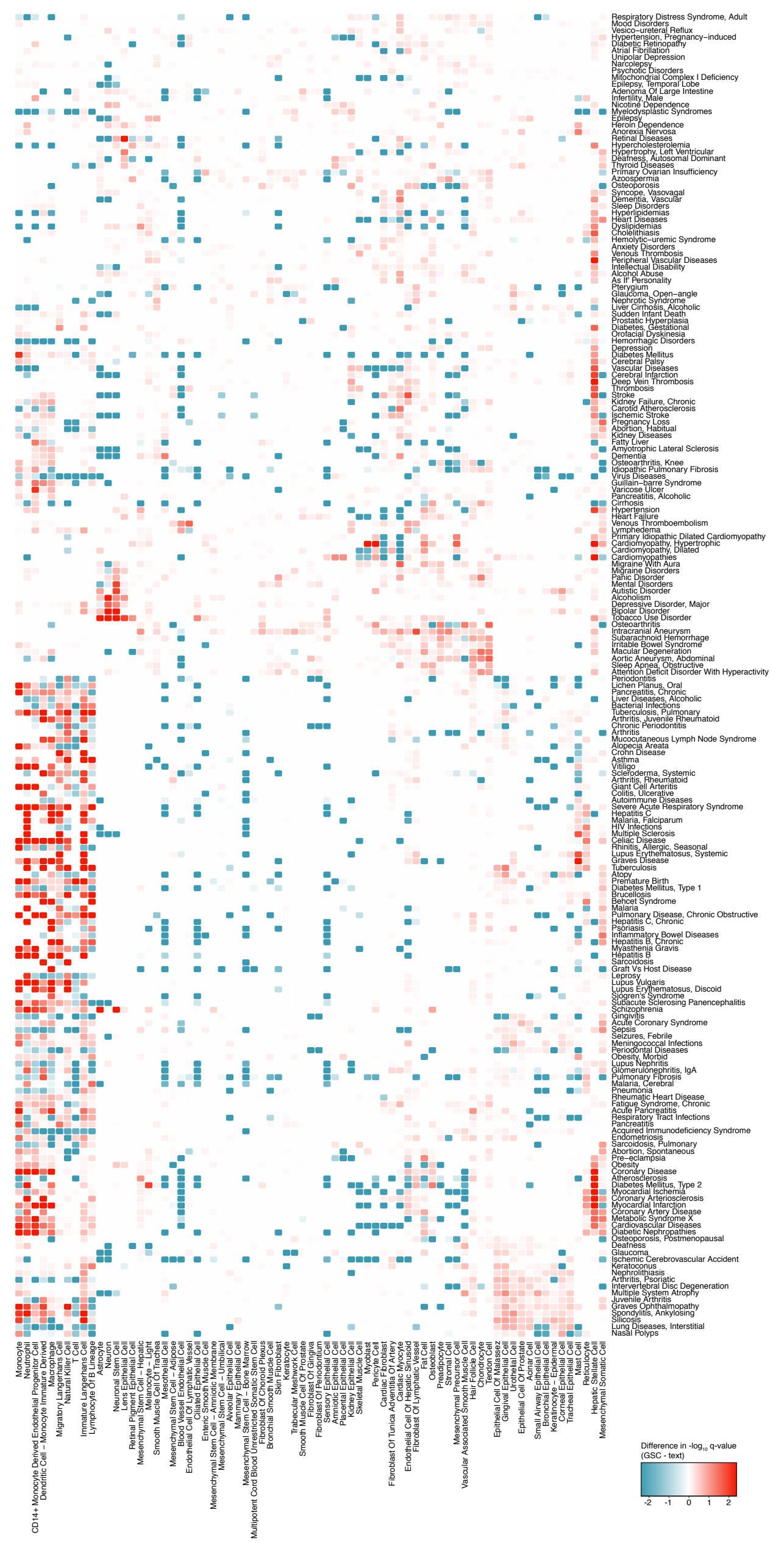

Supplement: Additional file 17 — Figure S6. Heat map comparing the disease–cell-type associations identified by the GSC method and text-mining. Differences are compared by first correcting the P values for multiple testing using the Benjamini–Hochberg procedure. The −log10 of the text-mined q value is then subtracted from the −log10 of the GSC method-computed q value. If the GSC method identifies the association as more significant, the corresponding cell is colored red. If text-mining identifies the association as more significant, the cell is colored blue. If the significance level is similar between methods, the cell is colored white. (PDF 3190 kb) [file 13073_2015_212_MOESM17_ESM.pdf]

## A) Associations supported by text-mining

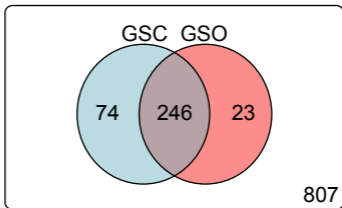

## B) Associations not supported by text-mining

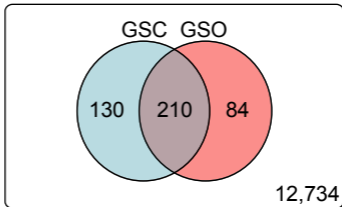

Supplement: Additional file 18 — Figure S7. Venn diagrams of the disease–cell-type associations (A) supported and (B) not supported by text-mining that are also identified by the GSC and GSO methods. Sets of associations were produced by applying an FDR cutoff of 10 % to the GSC, GSO and text-mining results. (PDF 677 kb) [file 13073_2015_212_MOESM18_ESM.pdf]

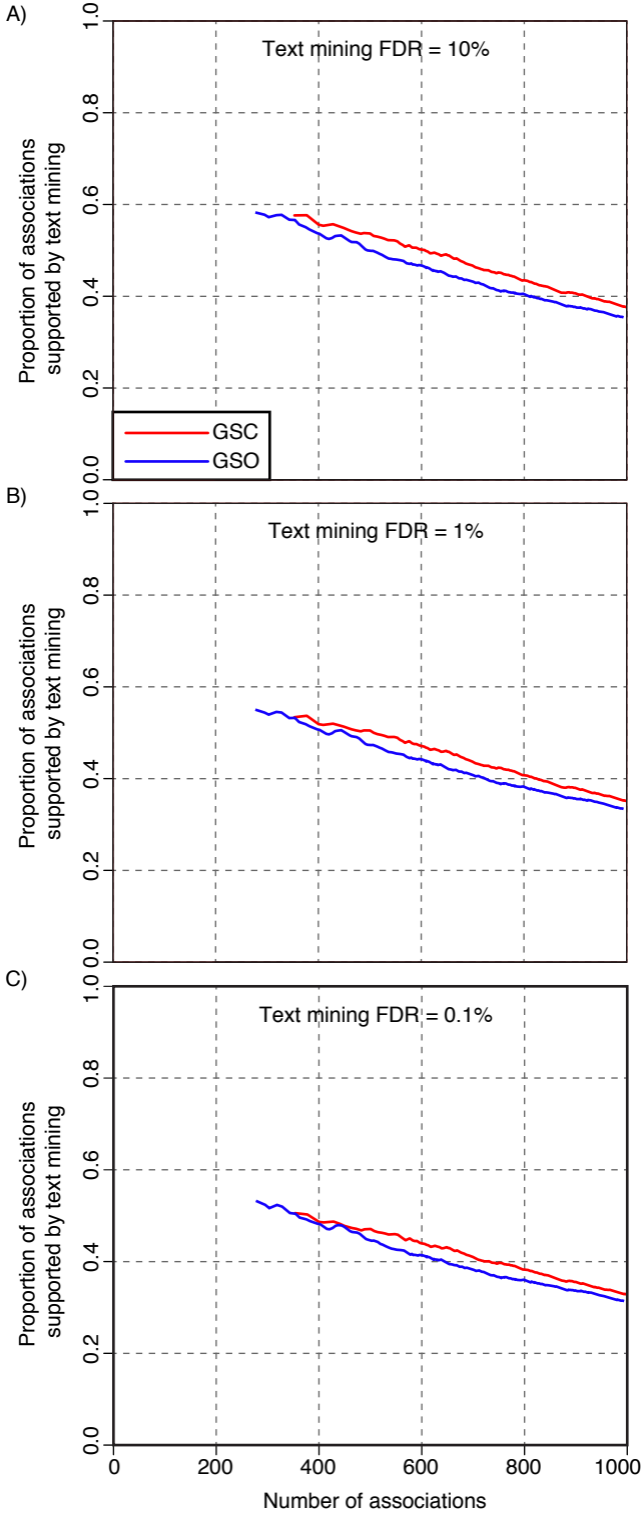

Supplement: Additional file 20 — Figure S8. The proportion of disease–cell-type associations identified by the GSC and GSO methods supported by text-mining at various cutoffs. Disease–cell-type associations were ranked by their q value. The number of associations represents the size of the set of the top-ranked disease–cell-type associations. To compute the proportion of disease–cell-type associations supported by text-mining, it was necessary to apply an FDR cutoff to the text-mining results. Here we use three cutoffs: (A) 10 %, (B) 1 % and (C) 0.1 %. The GSC method assigned 219 associations the lowest-possible P value, while the GSO method assigned 211 associations this P value. It was not possible to order these top-ranked associations and therefore the GSC and GSO curves start at 219 and 211 respectively. (PDF 706 kb) [file 13073_2015_212_MOESM20_ESM.pdf]

GSC associations supported by GSO

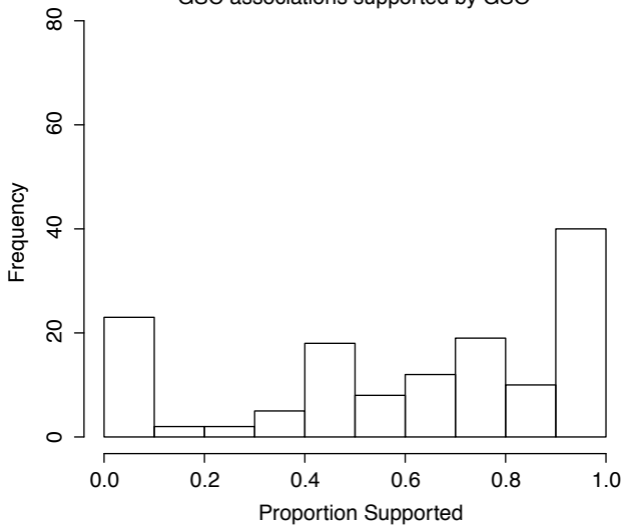

GSO associations supported by GSC

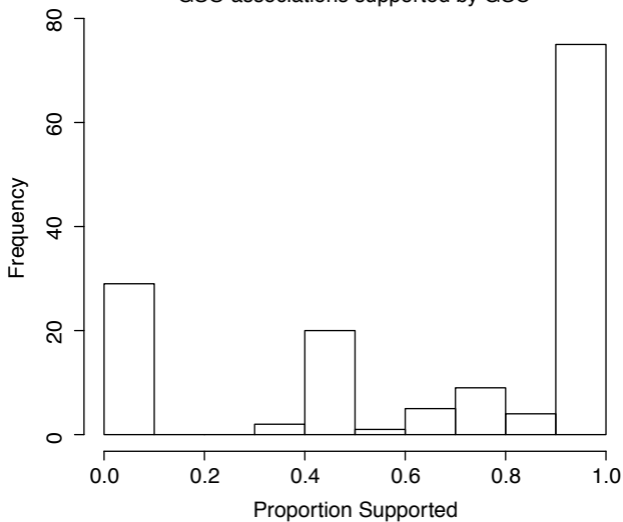

Supplement: Additional file 21 — Figure S9. Histograms showing the proportions of disease-associated cell types identified by one method supported by another. An FDR cutoff of 10 % was applied to the results of each method to produce sets of disease–cell-type associations. For each disease, the proportion of associations identified by method m 1 supported by method m 2 was computed by dividing the number of associations identified by method m 1 by the number of associations identified by both method m 1 and m 2. A value of 1 indicates that all associations identified by method m 1 are supported by method m 2 and a value of 0 indicates that no associations identified by method m 1 are supported by method m 2. Diseases where both methods identified no associated cell types were removed. There is a large amount of support between the GSC and GSO methods. For 64.0 % of diseases, at least 50.0 % of associations identified by the GSC method are supported by the GSO method. Similarly, for 64.8 % of diseases, at least 50.0 % of associations identified by the GSO method are supported by the GSC method. (PDF 1050 kb) [file 13073_2015_212_MOESM21_ESM.pdf]
